# Detection of Proximal Tubule Involvement by BK Polyomavirus in Kidney Transplant Recipients With Urinary Sediment Double-Immunostaining

**DOI:** 10.3389/fimmu.2020.582678

**Published:** 2020-09-23

**Authors:** Yang Huang, Xu-Tao Chen, Shi-Cong Yang, Hui-Fei Yang, Xiao-Tao Hou, Wen-Fang Chen, Jun Li, Rong-Hai Deng, Jin-Quan Luo, Jin-Yuan Wang, Xue Shen, Li-Zhong Chen, Chang-Xi Wang, Jiang Qiu, Gang Huang

**Affiliations:** ^1^Organ Transplant Center, The First Affiliated Hospital of Sun Yat-sen University, Guangzhou, China; ^2^Department of Pathology, The First Affiliated Hospital of Sun Yat-sen University, Guangzhou, China; ^3^Fuda Cancer Hospital, Jinan University, Guangzhou, China; ^4^Guangzhou KingMed Center for Clinical Laboratory Co., Ltd., Guangzhou, China; ^5^Zhongshan School of Medicine, Sun Yat-sen University, Guangzhou, China; ^6^Guangdong Provincial Key Laboratory of Organ Donation and Transplant Immunology, The First Affiliated Hospital, Sun Yat-sen University, Guangzhou, China; ^7^Guangdong Provincial International Cooperation Base of Science and Technology (Organ Transplantation), The First Affiliated Hospital, Sun Yat-sen University, Guangzhou, China

**Keywords:** kidney transplantation, BK polyomavirus, BKPyVAN, urinary sediment, double-immunostaining, proximal tubule

## Abstract

**Background:**

The extent and depth of BK polyomavirus (BKPyV) infection in renal allograft correlate with prognosis. This study was designed to evaluate the value of urinary sediment double-immunostaining for predicting BKPyV infection in proximal tubular epithelium.

**Materials and methods:**

A total of 76 urine sediment cell blocks, as well as the corresponding transplanted kidney tissues with BK polyomavirus associated-nephropathy (BKPyVAN), were evaluated by automatic double-immunostaining with anti-58-kDa Golgi protein (58K, a proximal renal tubular marker) + anti-SV40-T and anti-homogentisate 1, 2-dioxygenase (HGD, a renal tubular marker) + anti-SV40-T.

**Results:**

Immunohistochemical staining demonstrated that 58K was expressed in proximal tubular epithelium but not in distal tubular epithelium or transitional epithelium. Of the 76 patients, 28 (36.8%) had urinary 58K(+)/SV40-T(+) cells and HGD(+)/SV40-T(+) cells, 41 (53.9%) had only HGD(+)/SV40-T(+) cells, one (1.3%) had only 58K(+)/SV40-T(+) cells, and six (7.9%) had only 58K(−)/HGD(−)/SV40-T(+) cells. The presence of urinary 58K(+)/SV40-T(+) cells was correlated with BKPyV infection in proximal tubular epithelium (*P* < 0.001, *r* = 0.806). The mean extent of SV40-T staining was significantly more extensive in patients with urinary 58K(+)/SV40-T(+) cells than those without urinary 58K(+)/SV40-T(+) cells (21.4 vs. 12.0%, *P* < 0.001). The positive predictive value, negative predictive value, sensitivity, and specificity of urinary 58K(+)/SV40-T(+) cells for predicting BKPyV infection in proximal tubular epithelium were 89.7% (95% CI: 71.5–97.3%), 91.5% (95% CI: 78.7–97.2%), 86.7% (95% CI: 68.4–95.6%), and 93.5% (95% CI: 81.1–98.3%), respectively.

**Conclusion:**

Urinary sediment double-immunostaining with anti-58K and anti-SV40-T is valuable for predicting the extent and depth of BKPyV infection in renal allograft.

## Introduction

BK polyomavirus-associated nephropathy (BKPyVAN) is one of the severe complications resulting in renal graft dysfunction and graft loss (1, 2). BKPyVAN develops in 1 to 10% of kidney transplant recipients and results in renal allograft loss in as high as 50% of the involved recipients (3, 4).

During the progression of BKPyVAN, BK polyomavirus (BKPyV) usually infects the pelvis, collecting ducts, distal tubules, and subsequently proximal tubules (5–8). In some cases of severe infection, BKPyV can spread to the glomerular parietal epithelial cells (9–11). Drachenberg CB et al. reported that the extent of SV40-T staining was positively associated with BKPyVAN stages (12). Our previous study has demonstrated that BKPyV infection in glomerular parietal epithelial cells is a risk factor for accelerated renal graft failure, indicating the infection depth of BKPyV in allograft has an adverse effect on prognosis (9). Thus, if the depth and extent of BKPyV infection in allograft could be determined non-invasively, it may provide some useful information for risk stratification and prognosis evaluation of BKPyVAN. However, there are few such tests that can predict the extent and depth of intrarenal BKPyV infection.

Urine decoy cells detected by Papanicolaou staining refer to the epithelial cells shed after BKPyV infection (13). The negative predictive value (NPV) of decoy cells for predicting BKPyVAN is close to 100%, but it’s positive predictive value (PPV) is only 29% (14, 15). The main reason is that decoy cell can be derived from either renal tubular epithelium or ureteral and bladder transitional epithelium (16). Our previous study has proved that double-immunostaining with anti-homogentisate 1, 2-dioxygenase (anti-HGD) and anti-SV40-T on urinary sediment can effectively improve the PPV of decoy cell for diagnosing BKPyVAN (6). However, HGD is expressed in both proximal and distal tubular epithelium. Hence, HGD is not suitable for identifying the depth of BKPyV infection in renal allograft.

58-kDa Golgi protein (58K), also known as formimidoyltransferase cyclodeaminase (FTCD), is specifically expressed in proximal tubules but not in distal tubules or collecting ducts^1^. It can be reasonably hypothesized that the presence of 58K(+)/SV40-T(+) cells in urine indicates active BKPyV replication in proximal tubular epithelial cells. We performed this study to investigate the value of urinary 58K(+)/SV40-T(+) cells for predicting the extent and depth of BKPyV infection in renal allograft.

## Materials and Methods

### Study Population

This is a retrospective cross-sectional study conducted at a single center. A total of 76 kidney transplant recipients who were diagnosed with biopsy-proved BKPyVAN at our hospital from Aug 19, 2015 to Nov 25, 2019 were enrolled for analysis. All patients were diagnosed with BKPyVAN without receiving any treatment and had urinary decoy cell ≥1/10 high power fields (HPFs). Patients with repeated kidney transplantation or recurrent BKPyVAN were not enrolled. This study was approved by the Ethics Committee and the Research Board of our institution.

### Urine Cytology

A total of 10 mL of mid-morning urine was collected on the day of kidney biopsy. All patients were fasted for more than 8 h before collecting samples. No patient experienced oliguria or anuria in this study. After centrifugation (3000 rpm, 10 min), urine sediment was stored with buffer solution (LBP-1808003, Guangzhou). Cytology smears were made by a liquid-based thin-layer cell pelleter (Auto Cyto Prep System, LBP-2601, Guangzhou, Guangdong, China) and subsequently stained by the Papanicolaou stain method (17). Cells with intranuclear viral inclusions were defined as decoy cells and were counted as number per 10 HPFs (18).

### Virological Studies

BK polyomavirus viral load in urine and plasma samples was determined by a quantitative PCR (q-PCR) Detection Kit (SINOMED, Beijing, China), as described elsewhere (19). Assays were performed on an ABI Prism 7500 Sequence Detection system (Applied Biosystems, United States), according to the manufacturer’s instructions. PCR amplifications were performed in a 2 μL reaction volume that contained 5 μL of extracted DNA. Probes were designed to specifically detect the BKPyV and did not cross-react with sequences present in the related JC polyomavirus or SV40 polyomavirus, as per the manufacturer’s statement. The lower limit of quantitation was 1 × 10^3^ copies/mL for both plasma and urine.

### Pathological Diagnosis of BKPyVAN

Pathological lesions were scored according to the 2017 Banff criteria (20). The pathological diagnosis of BKPyVAN was confirmed by immunohistochemical (IHC) staining with an anti-SV40-T monoclonal antibody (mouse anti-SV40 large T antigen monoclonal antibody; Oncogene Research Products, Cambridge, MA, United States, cat # DP02, clone PAb 416), as previously described (21). The histological features of BKPyVAN were classified using the American Society of Transplantation (AST) schema and BKPyVAN was classified as A, B, and C based on the guidelines published by Hirsch et al. (22).

### Immunohistochemical Staining

Antigen retrieval was performed on 3 μm paraffin-embedded sections (pH 9.0, 100°C). Three primary antibodies were used, including anti-CD10 mouse monoclonal antibody (1:200, MAB-0668, Maixin, Fuzhou, Fujian, China), anti-58K rabbit monoclonal antibody (1:1500, Catalogue Number ab129005, Abcam, Cambridge, United Kingdom), and anti-HGD rabbit monoclonal antibody (1:3200, Catalogue Number ab131035, Abcam, Cambridge, United Kingdom). The chromogen for all 3 markers was diaminobenzidine (DAB). The IHC protocol for CD10, HGD, and 58K has been reported elsewhere (6, 23–25).

### Preparation of Cell Block

Patients were instructed to collect a total of 600 mL of fresh urine in a sterile bottle with 60 mL of 4% formaldehyde solution from 10:00 pm to 8:00 am. All patients were fasted for more than 8 h before collecting samples. No patient experienced oliguria or anuria in this study. The urine specimen was centrifuged for 10 min at 500 × *g*. The urine sediment cell blocks were prepared by the Anbiping kit (LBP Medicine Science and Technology Co., Ltd., Guangzhou, China, Batch number 1708001). All procedures were performed strictly in accordance with the instructions (6).

### Double-Immunohistochemical Staining

Double-immunostaining on 3 μm paraffin-embedded sections of transplanted kidney tissue and urinary sediment cell blocks were performed by using a fully automatic IHC staining machine (Leica Bond-Max, Germany). The main processes included anti-SV40 (mouse monoclonal antibody, 1:4,000, Catalogue Number DP02, Merck Millipore, United States), anti-HGD (rabbit monoclonal antibody, 1:3200, Catalogue Number ab131035, Abcam, Cambridge, United Kingdom), or anti-58K (rabbit monoclonal antibody, 1:1500, Catalogue Number ab129005, Abcam, Cambridge, United Kingdom), and finally Hematoxylin staining. The automatic double-immunostaining protocol was given in Supplementary Appendix S1.

### Statistical Analysis

Clinical data was expressed as count (percentage), or as mean ± SD. PPV, NPV, sensitivity, specificity, and 95% confidence intervals (CI) of urinary 58K(+)/SV40-T(+) cell for predicting BKPyV infection in proximal tubular epithelial cells were calculated. The receiver operator characteristic curve (ROC) of plasma BKPyV-DNA load was performed to predict BKPyV infection in proximal tubular epithelium. Correlation analysis between urinary 58K(+)/SV40-T(+) cells and BKPyV infection in proximal tubular epithelium was evaluated by a Chi-square test. Clinical data and various pathological damage indicators were compared by Student’s independent *t* test or the Mann-Whitney *U* test. IBM SPSS version 25 software (IBM Corporation, Somers, New York, United States) was used for statistical analyses. Values of *P* < 0.05 were considered statistically significant.

## Results

### Patient Characteristics

This study enrolled 76 patients with BKPyVAN, including 42 males and 34 females with a mean age of 41.3 years. A total of 76 cell blocks and 76 corresponding kidney tissues were evaluated by IHC staining. According to double IHC staining (anti-58K and anti-SV40-T) findings of kidney biopsies, all patients were subdivided into the BKPyV-infected proximal tubule group (*n* = 30) and the non-BKPyV-infected proximal tubule group (*n* = 46). Patient demographics and transplant characteristics are summarized in Table 1. The urine BKPyV-DNA load and plasma BKPyV-DNA load in the BKPyV-infected proximal tubule group were, respectively, higher than that in the non-BKPyV-infected proximal tubule group (*P* = 0.039, *P* = 0.049).

**TABLE 1:** Demographics and transplant characteristics of 76 patients with biopsy-proved BKPyVAN.

**Parameters**	**Total (*n* = 76)**	**Non-BKPyV-infected proximal tubule group (*n* = 46)**	**BKPyV-infected proximal tubule group (*n* = 30)**	***P***
**Gender**				0.842
Male (%)	42 (55.3)	25 (54.3)	17 (56.7)	
Female (%)	34 (44.7)	21 (45.7)	13 (43.3)	
Age (years)	41.3 ± 10.0	41.4 ± 9.5	41.2 ± 11.0	0.936
Time from transplant to BKPyVAN (years)	1.5 ± 1.3	1.7 ± 1.4	1.3 ± 1.1	0.324
**ESRD etiology**				0.520
Chronic glomerulonephritis (%)	28 (36.8)	17 (37.0)	11 (36.7)	
IgA nephropathy (%)	18 (23.7)	9 (19.6)	9 (30.0)	
Others (%)	30 (39.5)	20 (43.4)	10 (33.3)	
**Donor**				0.697
Living donor (%)	7 (9.2)	5 (10.9)	2 (6.7)	
Deceased donor (%)	69 (90.8)	41 (89.1)	28 (93.3)	
**Maintenance regimen**				0.416
Tac-MPA-Steroid (%)	75 (98.7)	45 (97.8)	30 (100.0)	
CsA-MPA-Steroid (%)	1 (1.3)	1 (2.2)	0 (0)	
Scr at baseline (μmol/L)	111.4 ± 37.1	111.9 ± 34.1	110.6 ± 41.9	0.886
Scr at biopsy (μmol/L)	207.4 ± 88.8	203.4 ± 86.4	213.4 ± 93.5	0.653
Decoy cells (/10 HPFs)	25.5 (IQR: 11.0–45.0)	25.0 (IQR: 12.0–43.0)	27.0 (IQR: 10.0–45.0)	0.503
Urine BKPyV-DNA load (copies/mL)	1.4 × 10^9^ (IQR: 5.7 × 10^7^–1.2 × 10^10^)	7.4 × 10^8^ (IQR: 3.7 × 10^7^–6.0 × 10^9^)	7.3 × 10^9^ (IQR: 1.1 × 10^8^–2.2 × 10^10^)	0.039
Plasma BKPyV-DNA load (copies/mL)	6.6 × 10^3^ (IQR: 4.1 × 10^2^–9.9 × 10^4^)	4.8 × 10^3^ (IQR: 0–4.2 × 10^4^)	2.3 × 10^4^ (IQR: 1.4 × 10^3^–2.0 × 10^5^)	0.049

*BKPyVAN, BK polyomavirus associated-nephropathy; BKPyV, BK polyomavirus; ESRD, end-stage renal disease; Tac, tacrolimus; CsA, cyclosporine A; MPA, mycophenolic acid; Scr, serum creatinine; HPFs, high power fields; IQR, interquartile range.*

### Urinary Decoy Cells

**FIGURE 1:**
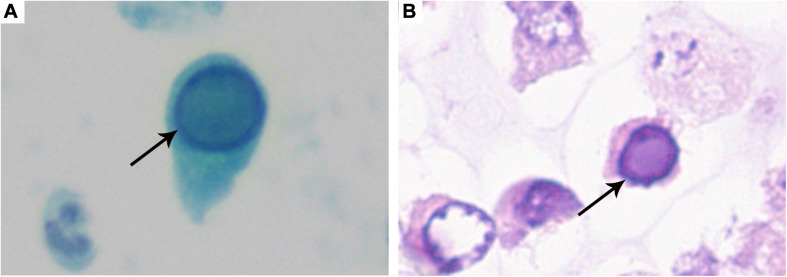
Typical decoy cell. **(A)** Decoy cell detected by Papanicolaou staining (arrow, ×1,000). **(B)** Decoy cell detected by Hematoxylin-Eosin staining (arrow, ×1,000).

Decoy cells were found in all 76 patients [median count 25.5/10 HPFs, interquartile range (IQR): 11.0–45.0]. Four classical morphological types of decoy cells had been described in our previous study (6). As shown in Figure 1, the classic decoy cell contained enlarged, basophilic, and homogeneous amorphous ground glass-like intra-nuclear inclusion bodies.

### CD10, 58K, and HGD Immunostaining

**FIGURE 2:**
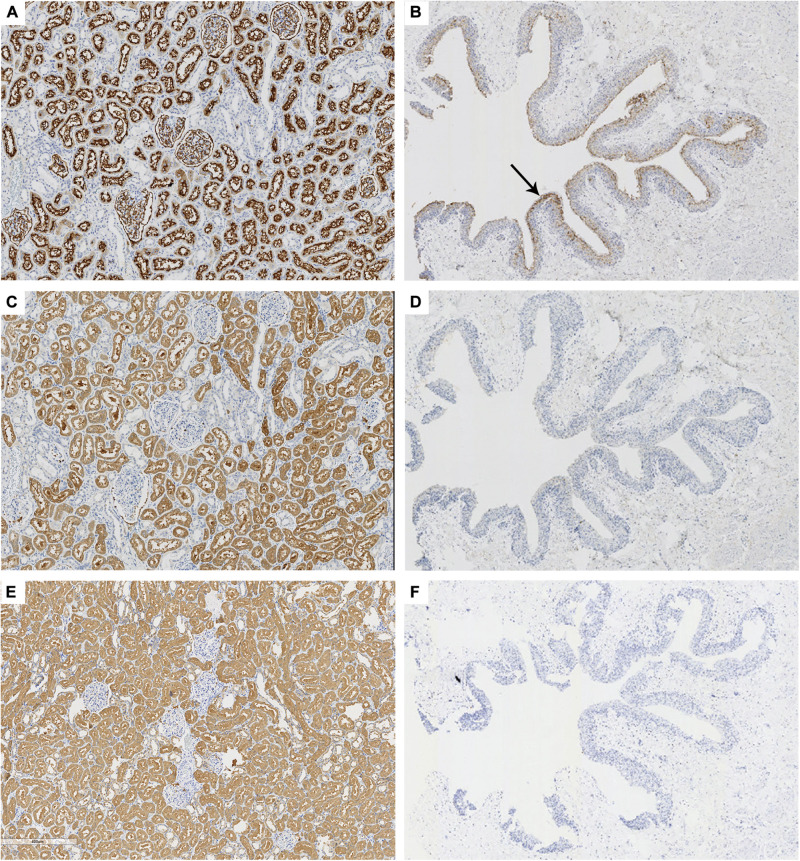
Immunohistochemical staining of CD10, 58K, and HGD in kidney and ureter tissues. Anti-CD10, anti-58K, and anti-HGD immunostaining in renal tissue (**A,C,E**, ×60) and transitional epithelium (**B,D,F**, ×18). Black arrow in **(B)** referred that CD10 was positive in the transitional epithelium (brown). 58-kDa Golgi protein (58K); homogentisate 1, 2-dioxygenase (HGD).

To confirm the specificity of 58K for proximal tubular epithelium, the serial sections of normal renal tissue were stained by anti-58K and anti-CD10, respectively. As shown in Figure 2, anti-CD10 immunostaining was positive only in the proximal tubular epithelium (brown, Figure 2A) and transitional epithelium (brown, Figure 2B), but not in the distal tubular epithelium or collecting ducts. In renal tissue, 58K was detected in the proximal tubular epithelium (brown, Figure 2C), and the staining extent and location were consistent with that of anti-CD10 immunostaining. Unlike CD10, 58K was not expressed in transitional epithelium (Figure 2D). HGD was detected in all tubular epithelium, including collecting ducts, proximal tubules, and distal tubules (brown, Figure 2E) but not in transitional epithelium (Figure 2F).

### Double-Immunostaining of Anti-58K/Anti-SV40-T and Anti-HGD/Anti-SV40-T on Kidney Tissue

**FIGURE 3:**
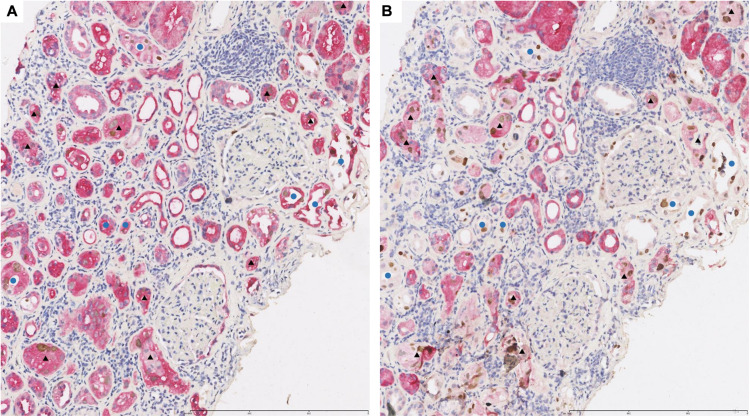
Representative pictures of double-immunostaining with SV40-T/HGD **(A)** or SV40-T/58K **(B)** in the same area from renal allograft with BKPyV infection. The infected proximal tubular epithelial cells expressed HGD/SV40-T (**A**, ×100) and 58K/SV40-T (**B**, ×100). Black triangles referred to the same proximal tubules infected by BKPyV in consecutive sections. The infected distal tubular epithelial cells expressed HGD/SV40-T (**A**, ×100) and SV40-T but not 58K (**B**, ×100). Blue circles referred to the same distal tubules infected by BKPyV in consecutive sections. 58-kDa Golgi protein (58K); homogentisate 1, 2-dioxygenase (HGD).

In transplanted kidney tissue with BKPyVAN, HGD was expressed in all renal tubular epithelial cells (Figure 3A) while 58K was expressed only in proximal renal tubular epithelial cells (Figure 3B). In Figures 3A,B, black triangles referred to the same proximal tubules infected by BKPyV in consecutive sections, and blue circles referred to the same distal tubules infected by BKPyV in consecutive sections. SV40-T can be detected in the nucleus of proximal tubular epithelium and distal tubular epithelium infected by BKPyV (Figures 3A,B). All tubular epithelial cells infected with BKPyV simultaneously expressed HGD and SV40-T (Figure 3A). Similarly, BKPyV-infected proximal tubular epithelial cells expressed 58K and SV40-T simultaneously. However, BKPyV-infected distal tubular and collecting duct epithelial cells expressed SV40-T but not 58K (Figure 3B). In normal renal tissue, double-immunostaining of anti-58K/anti-SV40-T and anti-HGD/anti-SV40-T showed that HGD was expressed in all tubular epithelium in medulla but neither 58K nor SV40-T did (Supplementary Appendix S2).

### Double-Immunostaining of Anti-58K/Anti-SV40-T and Anti-HGD/Anti-SV40-T on Urinary Sediment Cell Blocks

**FIGURE 4:**
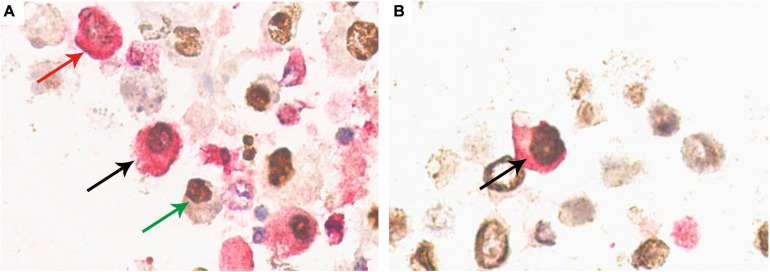
Confirmation of HGD(+)/SV40-T(+) cells and 58K(+)/SV40-T(+) cells on urinary sediment cell blocks by double-immunostaining (×400). **(A)** Double-immunostaining revealed cell with concurrent HGD expression (cytoplasm, red) and SV40-T expression (nuclei, brown) (black arrow), with the background showing single positiveness of either HGD expression in the cytoplasm (red arrow) or SV40-T expression in the nuclei (green arrow). **(B)** Double-immunostaining revealed a cell with 58K expression (cytoplasm, red) and SV40-T expression (nuclei, brown) (black arrow). 58-kDa Golgi protein (58K); homogentisate 1, 2-dioxygenase (HGD).

Typical decoy cells detected by double-immunostaining are presented in Figure 4. Double-immunostaining findings were qualitatively reported as either present (≥ one positive cell) or absent. In urinary sediment samples, the BKPyV-infected (positive SV40-T staining in nucleus) cells were categorized as HGD(+)/SV40-T(+) (Figure 4A, black arrow), 58K(+)/SV40-T(+) (Figure 4B, black arrow), and 58K(−)/HGD(−)/SV40-T(+) (Figure 4A, green arrow). Of the 76 patients, 28 (36.8%) had both 58K(+)/SV40-T(+) cells and HGD(+)/SV40-T(+) cells, 41 (53.9%) had only HGD(+)/SV40-T(+) cells, 1 (1.3%) had merely 58K(+)/SV40-T(+) cells, and 6 (7.9%) had only 58K(−)/HGD(−)/SV40-T(+) cells.

### Comparison of Pathological Findings Between Patients With Urinary 58K(+)/SV40-T(+) Cells and Those Without Urinary 58K(+)/SV40-T(+) Cells

Correlation analysis showed that the positivity of urinary 58K(+)/SV40-T(+) cells was strongly correlated with BKPyV infection in the proximal tubular epithelium (*P* < 0.001, *r* = 0.806). The mean extent of SV40-T staining in renal allograft was significantly more extensive in patients with urinary 58K(+)/SV40-T(+) cells than those without urinary 58K(+)/SV40-T(+) cells (21.4 vs. 12.0%, *P* < 0.001), while pathological stage of BKPyVAN, as well as various pathological Banff scores for tubulitis (t), interstitial inflammation (i), tubular atrophy (ct), and interstitial fibrosis (ci) were comparable (all *P* > 0.05, Table 2).

**TABLE 2:** Pathological characteristics between patients with urinary 58K(+)/SV40-T(+) cells and those without urinary 58K(+)/SV40-T(+) cells.

**Parameters**	**With urinary 58K(+)/SV40-T(+) cells (*n* = 29)**	**Without urinary 58K(+)/SV40-T(+) cells (*n* = 47)**	***P***
Extent of SV40-T staining (%)	21.4 ± 11.8	12.0 ± 7.5	<0.001
**Pathological Banff scores**			
t	1.8 ± 0.8	1.5 ± 0.7	0.127
i	1.1 ± 1.1	0.7 ± 0.8	0.520
ct	1.9 ± 0.7	1.7 ± 0.8	0.429
ci	1.9 ± 0.7	1.7 ± 0.7	0.272
**BKPyVAN stage (%)**			0.667
A	1 (3.4)	4 (8.5)	
B	25 (86.2)	38 (80.9)	
C	3 (10.3)	5 (10.6)	

*58K, 58-kDa Golgi protein; t, tubulitis; i, interstitial inflammation; ct, tubular atrophy; ci, interstitial fibrosis; BKPyVAN, BK polyomavirus associated-nephropathy.*

### Predictive Value of Urinary 58K(+)/SV40-T(+) Cells for Diagnosing BKPyV Infection in Proximal Tubular Epithelium

In all 76 patients with BKPyVAN, BKPyV infection in proximal tubular epithelial cells was confirmed in 30 (39.5%) patients, 25 (83.3%) of whom had urinary 58K(+)/SV40-T(+) cells and HGD(+)/SV40-T(+) cells, 3 (10.0%) had only HGD(+)/SV40-T(+) cells, 1 (3.3%) had only 58K(+)/SV40-T(+) cells, and 1 (3.3%) had only 58K(−)/HGD(−)/SV40-T(+) cells. The proportion of biopsy-proved BKPyV infection in proximal tubular epithelium was significantly higher in patients with urinary 58K(+)/SV40-T(+) cells than those without (26/29 vs. 4/47, *P* < 0.001). The PPV, NPV, sensitivity, and specificity of urinary 58K(+)/SV40-T(+) cells for predicting BKPyV infection in proximal tubular epithelium were 89.7% (95% CI: 71.5–97.3%), 91.5% (95% CI: 78.7–97.2%), 86.7% (95% CI: 68.4–95.6%), and 93.5% (95% CI: 81.1–98.3%), respectively.

### Performance of BKPyV Viremia for Predicting the Depth of BKPyV Infection in Renal Allograft

**FIGURE 5:**
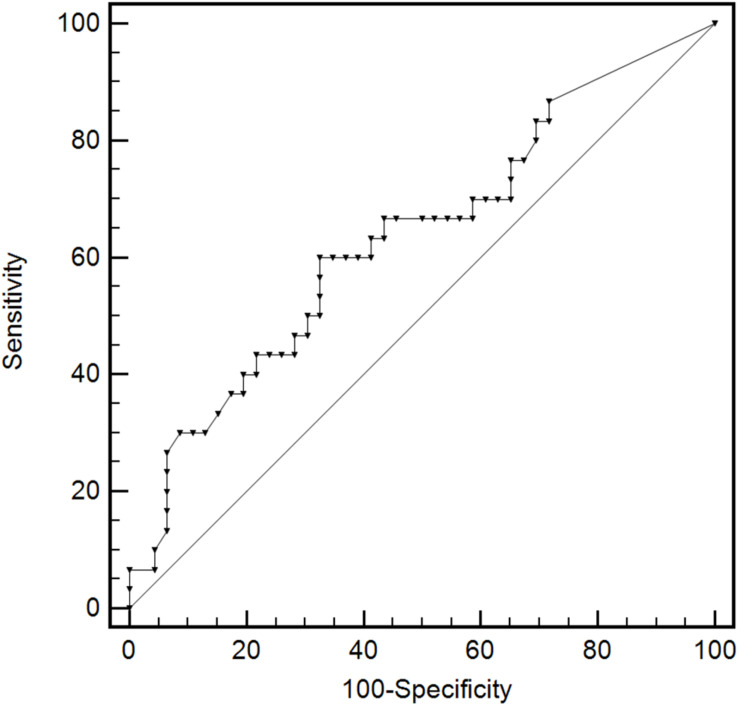
The ROC curve of plasma BKPyV-DNA load for predicting BKPyV infection in proximal tubular epithelium. Receiver operator characteristic curve (ROC).

The area under the ROC (AUC) of plasma BKPyV-DNA load for predicting BKPyV infection in proximal tubular epithelium was 0.634 (95% CI: 0.515–0.741), with an optimal cut-off value of 18000 copies/mL, a sensitivity of 60.0% (95% CI: 40.6–77.3%) and a specificity of 67.4% (95% CI: 52.0–80.5%) (Figure 5). The capacity of other plasma viral load thresholds for predicting BKPyV infection in proximal tubular epithelium was shown in Table 3. When grouped by different plasma viral load thresholds, the mean extent of SV40-T staining in renal allograft was significantly more extensive in patients with plasma BKPyV-DNA load ≥ 10^5^ copies/mL than those with plasma BKPyV-DNA load < 10^5^ copies/mL (20.1 vs. 14.1%, *P* = 0.027) and there was no significant difference in other pathological scores between the groups (Table 4).

**TABLE 3:** Plasma BKPyV-DNA load for predicting BKPyV infection in proximal tubular epithelium.

**Parameters**	**BKPyV-DNA load ≥ 10^3^ copies/mL**	**BKPyV-DNA load ≥ 10^4^ copies/mL**	**BKPyV-DNA load ≥ 10^5^ copies/mL**
Sensitivity (%)	80.0 (95% CI: 61.4–92.3%)	60.0 (95% CI: 40.6–77.3%)	33.3 (95% CI: 17.3–52.8%)
Specificity (%)	30.4 (95% CI: 17.7–45.8%)	63.0 (95% CI: 47.5–76.8%)	84.8 (95% CI: 71.1–93.7%)

*BKPyV, BK polyomavirus; CI, confidence intervals.*

**TABLE 4:** Pathological scores between patients with plasma BKPyV-DNA load ≥ the threshold and those with plasma BKPyV-DNA load < the threshold.

**Parameters**	**BKPyV-DNA load ≥ 10^3^ copies/mL (*n* = 57)**	**BKPyV-DNA load < 10^3^ copies/mL (*n* = 19)**	***P***	**BKPyV-DNA load ≥ 10^4^ copies/mL (*n* = 36)**	**BKPyV-DNA load < 10^4^ copies/mL (*n* = 40)**	***P***	**BKPyV-DNA load ≥ 10^5^ copies/mL (*n* = 19)**	**BKPyV-DNA load < 10^5^ copies/mL (*n* = 57)**	***P***
Extent of SV40-T staining (%)	15.8 ± 9.2	15.0 ± 13.7	0.781	16.7 ± 8.9	14.6 ± 11.6	0.366	20.1 ± 8.9	14.1 ± 10.5	0.027
**Pathological Banff scores**									
t	1.7 ± 0.7	1.3 ± 0.7	0.070	1.7 ± 0.6	1.5 ± 0.8	0.099	1.9 ± 0.7	1.5 ± 0.7	0.055
i	0.8 ± 0.9	0.7 ± 0.9	0.681	0.9 ± 0.9	0.7 ± 0.9	0.254	1.4 ± 0.9	0.6 ± 0.8	0.004
ct	1.8 ± 0.8	1.7 ± 0.7	0.819	1.7 ± 0.7	1.9 ± 0.8	0.313	1.9 ± 0.6	1.7 ± 0.8	0.476
ci	1.8 ± 0.8	1.7 ± 0.6	0.605	1.8 ± 0.7	1.9 ± 0.7	0.613	1.9 ± 0.6	1.8 ± 0.7	0.642
**BKPyVAN stage (%)**			0.649			0.366			0.649
A	4 (7.0)	1 (5.3)		3 (8.3)	2 (5.0)		1 (5.3)	4 (7.0)	
B	46 (80.7)	17 (89.4)		31 (86.1)	32 (80.0)		17 (89.4)	46 (80.7)	
C	7 (12.3)	1 (5.3)		2 (5.6)	6 (15.0)		1 (5.3)	7 (12.3)	

*BKPyV, BK polyomavirus; t, tubulitis; i, interstitial inflammation; ct, tubular atrophy; ci, interstitial fibrosis; BKPyVAN, BK polyomavirus associated-nephropathy.*

## Discussion

This study showed that urinary sediment double IHC staining with anti-58K and anti-SV40-T can identify BKPyV-infected proximal tubular epithelial cells with a sensitivity of 86.7% and specificity of 93.5%. This non-invasive method can be utilized to predict the extent and depth of BKPyV infection in the transplanted kidney and thus may predict the progression of BKPyVAN.

58-kDa Golgi protein is a bifunctional enzyme that catalyzes the conversion from tetrahydrofolate to 5, 10-methenyl tetrahydrofolate (26). In the urinary system, 58K was reported and confirmed by our study to be exclusively expressed in proximal tubular epithelial cells (see text footnote 1). Thus, it is reasonable to recognize 58K as a specific marker of proximal tubular epithelial cells.

Studies have shown that the extent of SV40-T immunostaining and the polyomavirus replication/load level (pvl) in the transplanted kidney had a negative impact on allograft function and prognosis (27–30). The Banff working group found that higher intrarenal pvl and Banff ci scores significantly correlated with more advanced BKPyVAN stage, worse renal allograft function, and a higher risk of graft loss (28). Our results showed that the extent of intrarenal SV40-T immunostaining was significantly larger in patients with urinary 58K(+)/SV40-T(+) cells than those without urinary 58K(+)/SV40-T(+) cells. This suggests that urinary sediment double IHC staining with anti-58K and anti-SV40-T can be used to predict the extent of BKPyV infection in a renal allograft.

It is worth noting that some studies have suggested that BKPyV spreads in the urinary epithelium through a retrograde pathway, that is, in the order of collecting tube, distal tubule, proximal tubule, and finally glomerular parietal epithelium (5–8). Other studies have found that early stage of BKPyVAN was focally distributed in the kidney and mainly in the medulla area (1, 28, 30, 31). A study by Yamanaka K et al. also showed that BKPyV usually infected collecting tubules and distal tubules at an early stage and involved proximal tubules at an advanced stage (32). Similarly, the findings from the Banff working group showed that BKPyV only infected the medulla at class 1 of BKPyVAN but extended from medulla to cortex at class 2 and class 3 of BKPyVAN (28). Our previous study proved that BKPyV involvement in glomerular parietal epithelial cells is an independent risk factor for renal allograft failure (9). The results of this study showed that 69 (90.8%) of 76 biopsy-proved BKPyVAN recipients had urinary HGD(+)/SV40-T(+) cells, which was consistent with our previous study (6). Besides, in the non-BKPyV-infected proximal tubule group, 41 (89.1%) patients had HGD(+)/SV40-T(+) cells. Therefore, HGD can effectively predict BKPyVAN, but fails to distinguish whether BKPyV infects proximal tubular epithelium or not. On this basis, we carried out this study to further prove the diagnostic value of HGD(+)/SV40-T(+) combined with 58K(+)/SV40-T(+) cells for BKPyVAN with proximal tubular epithelial cells infection. The PPV and NPV of 58K(+)/SV40-T(+) cells in urinary sediment block were as high as 89.7 and 91.5%, respectively, in predicting BKPyV infection in proximal tubular epithelial cells. Hence, the combined utilization of double-immunostaining with anti-HGD/anti-SV40-T and anti-58K/anti-SV40-T can accurately predict the depth of BKPyV infection and thereby assisting in risk stratification and treatment strategy.

In all 76 patients with BKPyVAN, BKPyV infection in proximal tubular epithelial cells was confirmed in 30 (39.5%) patients, 25 (83.3%) of whom had urinary 58K(+)/SV40-T(+) cells and HGD(+)/SV40-T(+) cells, 3 (10.0%) had only HGD(+)/SV40-T(+) cells, 1 (3.3%) had only 58K(+)/SV40-T(+) cells. Therefore, we recommend combing two double IHC staining methods of 58K/SV40-T and with HGD/SV40-T for diagnosing BKPyVAN. In addition, one patient was diagnosed with biopsy-proved BKPyV infection in proximal tubular epithelial cells, but only had 58K(−)/HGD(−)/SV40-T(+) cells in urine. This may be due to the destruction of the cell structure of the shed renal tubular cells during the process of excretion, resulting in a part of naked nuclear cells, which makes the cytoplasm unable to be stained. Moreover, in all 76 patients with BKPyVAN, non-BKPyV infection in proximal tubular epithelial cells was confirmed in 46 (60.5%) patients, 3 (6.5%) of whom had urinary 58K(+)/SV40-T(+) cells. We believe that due to kidney biopsy sample errors, it may lead to misdiagnosis of BKPyVAN. Thus, this also shows that double IHC staining with anti-58K and anti-SV40-T can make up for the shortcomings of renal biopsy.

The study performed by Hirsch H et al. showed that the possibility of BKPyVAN increased significantly when the threshold value of plasma BKPyV-DNA was increased to 10000 copies/mL (33). Some studies found that the higher plasma BKPyV-DNA load, the higher intrarenal BKPyV load and pathological grade of BKPyVAN (12, 34). Conversely, other studies have shown that plasma BKPyV load didn’t correlate the pathological grade of BKPyVAN (35–37). Our results showed that the AUC of plasma BKPyV-DNA load for predicting BKPyV infection in proximal tubular epithelial cells was as low as 0.634, with an optimal cut-off value of 18000 copies/mL, a sensitivity of 60.0% and a specificity of 67.4%. Although the specificity increased as the threshold increased, the sensitivity decreased obviously. The ability of plasma viral load to predict BKPyV infection in proximal tubular epithelium was limited. On the contrary, the sensitivity and specificity of urinary 58K(+)/SV40-T(+) cells for predicting BKPyV infection in proximal tubular epithelial cells were as high as 86.7 and 93.5%, respectively. These results suggested that urine sediment double-immunostaining was more suitable than BKPyV viremia for predicting the depth of BKPyV infection.

The quantitative urine BKPyV-Haufen test has been reported as a promising method to diagnose the degree of injury and pathological stage of BKPyVAN (37, 38). However, BKPyV-Haufen test has not been verified by multiple centers, just like our method. We believe that these technologies can be used in clinical practice after being verified by large sample research.

In summary, urinary sediment double-immunostaining with anti-58K and anti-SV40-T can help identify BKPyV infection in proximal tubular epithelial cells and thereby predicting the extent and depth of BKPyV infection in the transplanted kidney.

## Data Availability Statement

The raw data supporting the conclusions of this article will be made available by the authors, without undue reservation.

## Ethics Statement

The studies involving human participants were reviewed and approved by the Ethics Committee and the Research Board, the First Affiliated Hospital of Sun Yat-sen University [No. (2019)220]. The patients/participants provided their written informed consent to participate in this study.

## Author Contributions

YH, X-TC, GH, and JQ participated in research design. YH, S-CY, and W-FC participated in the performance of the research. X-TH and H-FY contributed to new reagents or analytic tools. J-QL, R-HD, JL, and J-YW contributed to collection and assembly of data. YH, X-TC, and S-CY participated in data analysis. All authors participated in the writing of the manuscript and contributed to final approval of manuscript.

## Conflict of Interest

X-TH was employed by Guangzhou KingMed Center for Clinical Laboratory Co., Ltd. The remaining authors declare that the research was conducted in the absence of any commercial or financial relationships that could be construed as a potential conflict of interest.

## Footnotes

1 https://www.proteinatlas.org/ENSG00000160282-FTCD/tissue

## Abbreviations

58K: 58-kDa Golgi protein
AST: American Society of Transplantation
AUC: area under the ROC
BKPyV: BK polyomavirus
BKPyVAN: BK polyomavirus-associated nephropathy
CI: confidence intervals
FTCD: formimidoyltransferase cyclodeaminase
HGD, homogentisate 1: 2-dioxygenase
HPFs: high power fields
IHC: immunohistochemical
IQR: interquartile range
NPV: negative predictive value
PPV: positive predictive value
pvl: polyomavirus replication/load level
q-PCR: quantitative PCR
ROC: receiver operator characteristic curve.
