# Supplementary material for: Detection of Proximal Tubule Involvement by BK Polyomavirus in Kidney Transplant Recipients With Urinary Sediment Double-Immunostaining
Source: Front Immunol. 2020 Sep 23;11:582678. doi: 10.3389/fimmu.2020.582678 (PMC7539630; doi:10.3389/fimmu.2020.582678)
Supplement: Supplementary file 1 [file Table_1.DOCX]

Supplementary Material

**Appendix S1 The automatic double staining protocol (Sequential double staining) was set as follows.**

- **Part 1 Equivalent to the following operating procedure step V) to step XIX) (IHC protocol, Leica, Microsystems, batch number 62798)**
  - Marker 1, anti-SV40-T mouse monoclonal antibody, 15 min,
  - Post Primary, 8 min,
  - Polymer, 8 min,
  - Peroxide Block, 5 min,
  - Mixed DAB Refine, 5 s,
  - Mixed DAB Refine, 5 min.
- **Part 2 Equivalent to the following operating procedure step XXII) to step XXVI) (IHC protocol, Leica, Microsystems, batch number 65131)**
  - Marker 2, anti-58K rabbit monoclonal antibody or anti-HGD rabbit monoclonal antibody, 15 min,
  - Post Primary alkaline phosphatase (AP) 20 min,
  - Polymer AP 30 min,
  - Mixed Red Refine 10 min,
  - Mixed Red Refine 5 min,
  - Hematoxylin 6 min.

The detailed procedure of double-immunostaining (anti-SV40-T + anti-HGD or anti-SV40-T + anti-58K) was as follows

1. Deparaffinizing with Bond solution,
2. Rinsing with 100% alcohol for 1 min, 3 times,
3. Rinsing with Bond wash solution for 1 min, 3 times,
4. Antigen retrieval with Bond epitope retrieval solution for 20 min (pH 6.0, 100 ℃ for HGD, or pH 9.0, 100 ℃ for 58K), followed by washing with wash buffer for 1 min, 3 times,
5. Incubating with anti-SV40-T antibody, 15 min (37 ℃),
6. Rinsing with Bond wash solution for 1 min, 3 times,
7. Post-primary, 8 min,
8. Rinsing with Bond wash solution for 1 min, 3 times,
9. Polymer, 8 min,
10. Rinsing with Bond wash solution for 1 min, 3 times,
11. Peroxide block, 5 min,
12. Rinsing with Bond wash solution for 1 min, 3 times,
13. Mixed DAB Refine, 5 s,
14. Mixed DAB Refine, 5 min,
15. Rinsing with Bond deionized water solution for 1 min, 3 times,
16. Rinsing with Bond wash solution for 1 min, 3 times,
17. Incubating with the anti-HGD antibody, 15 min (37 ℃),
18. Rinsing with Bond wash solution for 1 min, 3 times,
19. Post Primary AP, 20 min,
20. Rinsing with Bond wash solution for 1 min, 3 times,
21. Post Primary AP, 20 min,
22. Rinsing with Bond wash solution for 1 min, 3 times,
23. Polymer AP, 30 min,
24. Rinsing with Bond wash solution for 1 min, 3 times,
25. Mixed Red Refine, 10 min,
26. Mixed Red Refine, 5 min,
27. Rinsing with Bond deionized water solution for 1 min, 3 times,
28. Rinsing with Bond wash solution for 1 min, 3 times,
29. Hematoxylin, 6 min,
30. Rinsing with Bond deionized water solution for 1 min, 3 times,
31. Air drying,
32. Transparentizing with xylene,
33. Mounted with resin.

**Appendix S2 Double-immunostaining in normal renal tissue without BKPyVAN. HGD was expressed in all tubular epithelium in medulla but SV40-T did not (A × 60). Neither 58K nor SV40-T was expressed in all tubular epithelium in medulla (B × 60).**

**
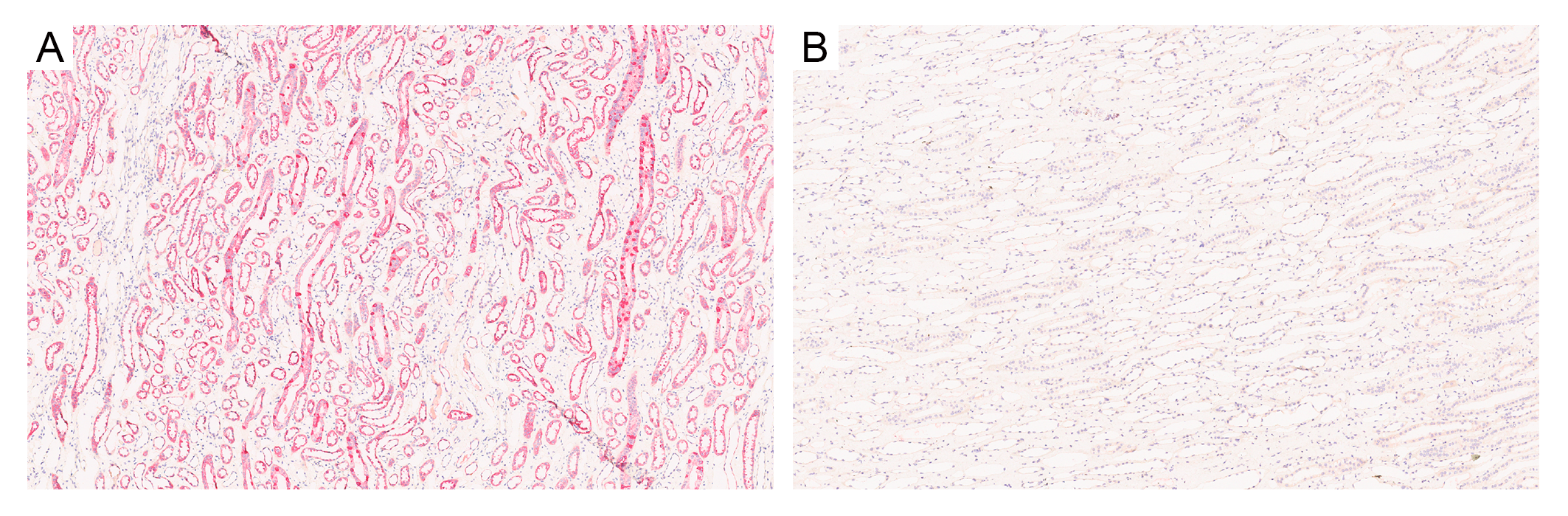
**
